# 38. Remdesivir Treatment in Patients Hospitalized with COVID-19: A Comparative Analysis of In-Hospital All-Cause Mortality

**DOI:** 10.1093/ofid/ofab466.038

**Published:** 2021-12-04

**Authors:** Essy Mozaffari, Aastha Chandak, Zhiji Zhang, Shuting Liang, Mark Thrun, Robert L Gottlieb, Daniel R Kuritzkes, Paul Sax, David Wohl, Roman Casciano, Paul Hodgkins, Richard Haubrich

**Affiliations:** 1 Gilead Sciences, Foster City, California; 2 Certara, New York, New York; 3 Baylor University Medical Center, Dallas, Texas; 4 Brigham and Women's Hospital, Cambridge, Massachusetts; 5 UNC School of Medicine, Chapel Hill, North Carolina

## Abstract

**Background:**

Remdesivir (RDV) reduced time to recovery and mortality in some subgroups of hospitalized patients in the NIAID ACTT-1 RCT compared to placebo. Comparative effectiveness data in clinical practice are limited.

**Methods:**

Using the Premier Healthcare Database, we compared survival for adult non-mechanically ventilated hospitalized COVID-19 patients between Aug-Nov 2020 and treated with RDV within 2 days of hospitalization vs. those who did not receive RDV. Preferential within-hospital propensity score matching with replacement was used. Patients were matched on baseline O_2_ and 2-month admission period and were excluded if discharged within 3 days of RDV initiation (to exclude anticipated discharges/transfers within 72 hrs consistent with ACTT-1 study). Time to 14- and 28-day mortality was examined separately for patients on high-flow/non-invasive ventilation (NIV), low-flow, and no supplemental O_2_ using Cox Proportional Hazards models.

**Results:**

RDV patients (n=27,559) were matched to unique non-RDV patients (n=15,617) (**Fig 1**). The two groups were balanced; median age 66 yrs and 73% white (RDV); 68 yrs and 74% white (non-RDV), and 55% male. At baseline, 21% required high-flow O_2_, 50% low-flow O_2_, and 29% no O_2_, overall.

Mortality in RDV patients was 9.6% and 13.8% on days 14 and 28, respectively. For non-RDV patients, mortality was 14.0% and 17.3% on days 14 and 28, respectively. Kaplan-Meier curves for time to mortality are shown in **Fig 2**. After adjusting for baseline and clinical covariates, RDV patients on no O_2_ and low-flow O_2_ had a significantly lower risk of death within 14 days (no O_2_, HR: 0.69, 95% CI: 0.57—0.83; low-flow, HR: 0.67, 95% CI: 0.59—0.77) and 28 days (no O_2_, HR: 0.80, 95% CI: 0.68—0.94; low-flow, HR: 0.76, 95% CI: 0.68—0.86). Additionally, RDV patients on high-flow O_2_/NIV had a significantly lower risk of death within 14 days (HR: 0.81, 95% CI: 0.70—0.93); but not at 28 days (**Fig 3**).

Fig 1. Study Population

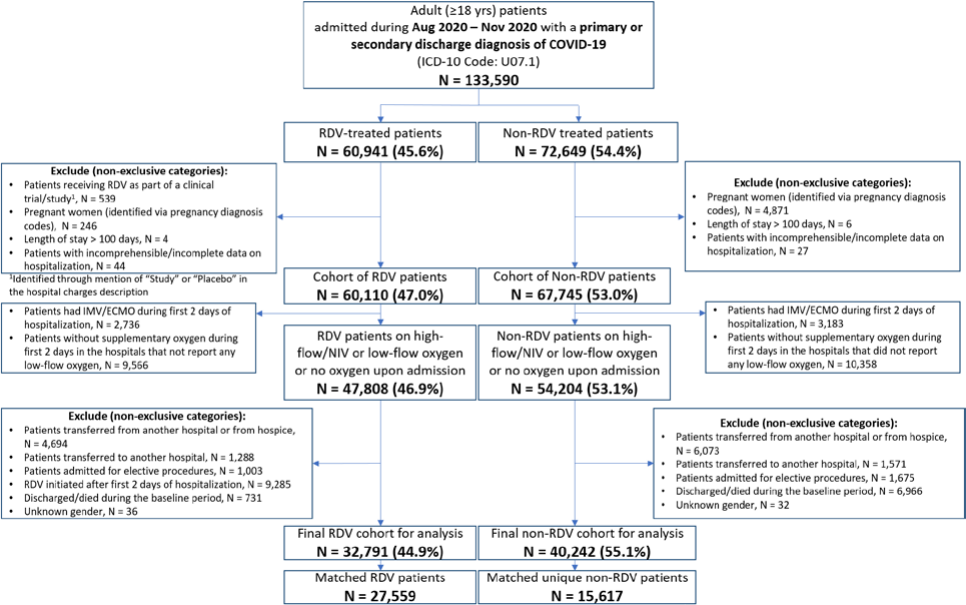

Fig 2. Kaplan-Meier curves among matched patients hospitalized for COVID-19, August-November 2020

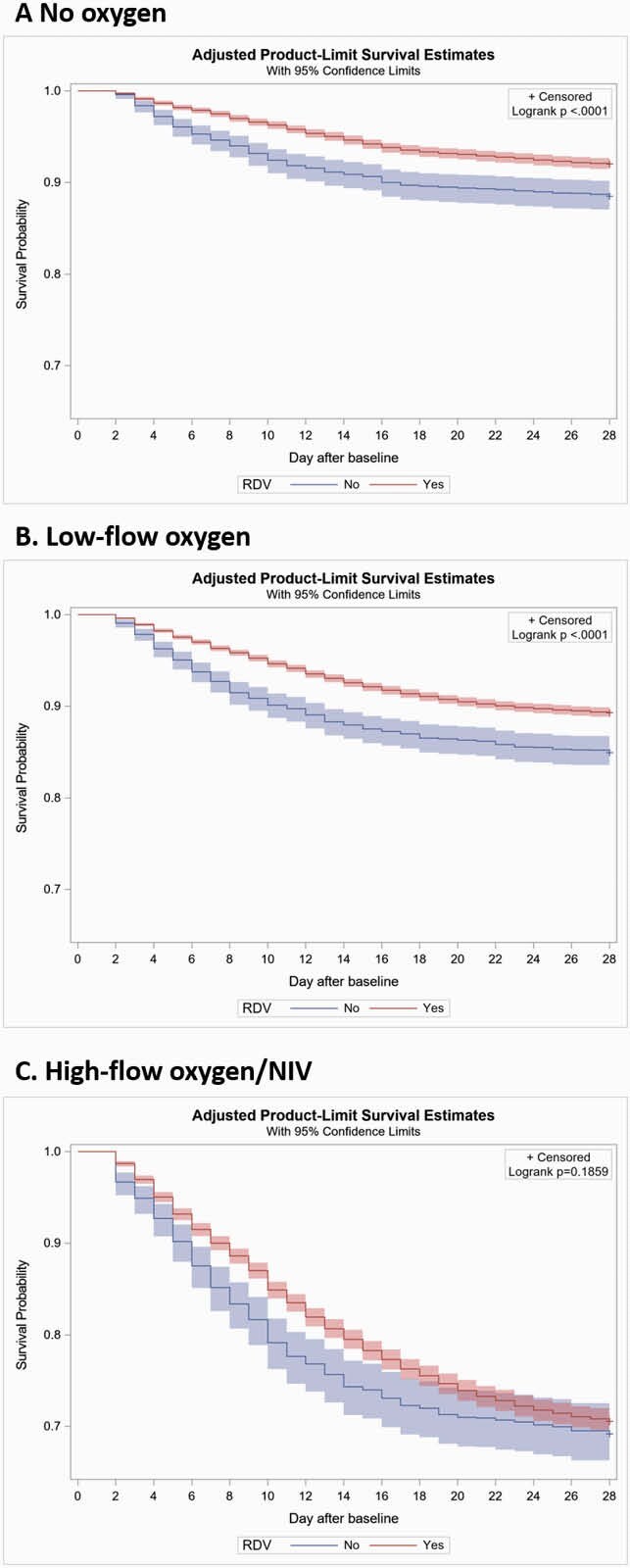

Fig 3. Cox proportional hazard model* for time to mortality among matched patients hospitalized for COVID-19, August-November 2020

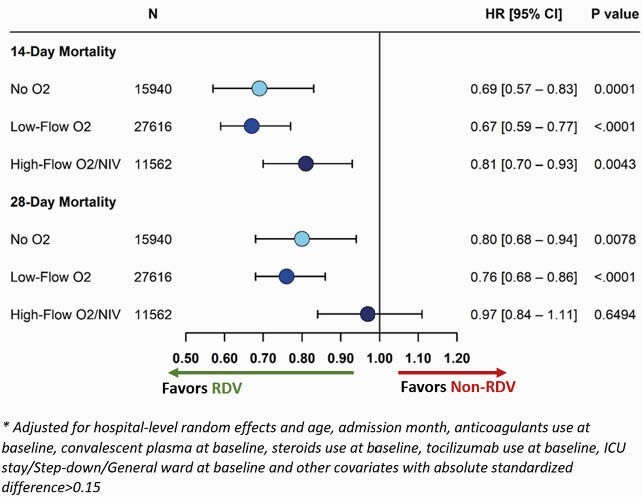

**Conclusion:**

In this large study of patients in clinical care hospitalized with COVID-19, we observed a significant reduction of mortality in RDV vs. non-RDV treated patients in those on no O_2_ or low-flow O_2_. Mortality reduction was also seen in patients on high-flow O_2_ at day 14, but not day 28. These data support the use of RDV early in the course of COVID-19 in hospitalized patients.

**Disclosures:**

**Essy Mozaffari, PharmD, MPH, MBA**, **Gilead Sciences** (Employee, Shareholder) **Aastha Chandak, PhD**, **Gilead Sciences** (Other Financial or Material Support, Employee of Certara (contracted by Gilead to conduct this study)) **Zhiji Zhang, MS**, **Gilead Sciences** (Other Financial or Material Support, Employee of Certara (contracted by Gilead to conduct this study)) **Shuting Liang, MPH**, **Gilead Sciences** (Employee) **Mark Thrun, MD**, **Gilead Sciences** (Employee, Shareholder) **Robert L. Gottlieb, MD**, **Eli Lilly** (Scientific Research Study Investigator, Advisor or Review Panel member)**Gilead Sciences** (Scientific Research Study Investigator, Advisor or Review Panel member, Other Financial or Material Support, Gift in kind to Baylor Scott and White Research Institute for NCT03383419)**GSK** (Advisor or Review Panel member)**Johnson and Johnson** (Scientific Research Study Investigator)**Kinevant** (Scientific Research Study Investigator)**Roche/Genentech** (Scientific Research Study Investigator) **Daniel R. Kuritzkes, MD**, **Abpro** (Consultant)**Atea** (Consultant, Scientific Research Study Investigator)**Decoy** (Consultant)**Gilead Sciences** (Consultant, Grant/Research Support)**GSK** (Consultant)**Janssen** (Consultant)**Merck** (Consultant, Grant/Research Support)**Novartis** (Scientific Research Study Investigator)**Rigel** (Consultant)**ViiV** (Consultant, Grant/Research Support) **Paul Sax, MD**, **Gilead Sciences** (Consultant, Grant/Research Support)**Janssen** (Consultant)**Merck** (Consultant, Research Grant or Support)**ViiV** (Consultant, Research Grant or Support) **David Wohl, MD**, **Gilead Sciences** (Consultant, Grant/Research Support, Advisor or Review Panel member)**Janssen** (Consultant, Advisor or Review Panel member)**Merck** (Consultant, Grant/Research Support, Advisor or Review Panel member)**ViiV** (Consultant, Grant/Research Support, Advisor or Review Panel member) **Roman Casciano, M.Eng**, **Gilead Sciences** (Other Financial or Material Support, Employee of Certara (contracted by Gilead to conduct this study)) **Paul Hodgkins, PhD, MSc**, **Gilead Sciences** (Employee, Shareholder) **Richard Haubrich, MD**, **Gilead Sciences** (Employee, Shareholder)

